# KVFinder-web: a web-based application for detecting and characterizing biomolecular cavities

**DOI:** 10.1093/nar/gkad324

**Published:** 2023-05-04

**Authors:** João V S Guerra, Helder V Ribeiro-Filho, José G C Pereira, Paulo S Lopes-de-Oliveira

**Affiliations:** Brazilian Biosciences National Laboratory (LNBio), Brazilian Center for Research in Energy and Materials (CNPEM), Campinas, São Paulo, 13083-100, Brazil; Graduate Program in Pharmaceutical Sciences, Faculty of Pharmaceutical Sciences, University of Campinas, Campinas, São Paulo, 13083-871, Brazil; Brazilian Biosciences National Laboratory (LNBio), Brazilian Center for Research in Energy and Materials (CNPEM), Campinas, São Paulo, 13083-100, Brazil; Brazilian Biosciences National Laboratory (LNBio), Brazilian Center for Research in Energy and Materials (CNPEM), Campinas, São Paulo, 13083-100, Brazil; Brazilian Biosciences National Laboratory (LNBio), Brazilian Center for Research in Energy and Materials (CNPEM), Campinas, São Paulo, 13083-100, Brazil; Graduate Program in Pharmaceutical Sciences, Faculty of Pharmaceutical Sciences, University of Campinas, Campinas, São Paulo, 13083-871, Brazil

## Abstract

Molecular interactions that modulate catalytic processes occur mainly in cavities throughout the molecular surface. Such interactions occur with specific small molecules due to geometric and physicochemical complementarity with the receptor. In this scenario, we present KVFinder-web, an open-source web-based application of parKVFinder software for cavity detection and characterization of biomolecular structures. The KVFinder-web has two independent components: a RESTful web service and a web graphical portal. Our web service, KVFinder-web service, handles client requests, manages accepted jobs, and performs cavity detection and characterization on accepted jobs. Our graphical web portal, KVFinder-web portal, provides a simple and straightforward page for cavity analysis, which customizes detection parameters, submits jobs to the web service component, and displays cavities and characterizations. We provide a publicly available KVFinder-web at https://kvfinder-web.cnpem.br, running in a cloud environment as docker containers. Further, this deployment type allows KVFinder-web components to be configured locally and customized according to user demand. Hence, users may run jobs on a locally configured service or our public KVFinder-web.

## Graphical Abstract

**Graphical Abstract:**
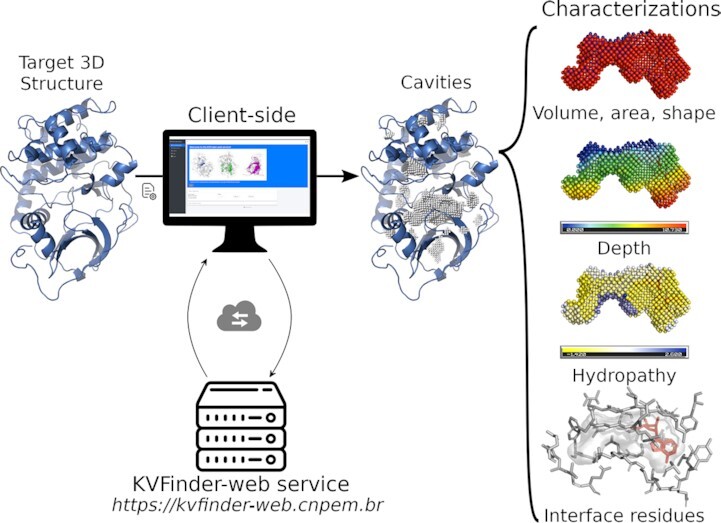
KVFinder-web: A web-based application for cavity detection and characterization of any type of biomolecular structure.

## INTRODUCTION

Biomolecules, such as proteins, perform biological processes by interacting with other molecules at binding sites (1,2). These sites are usually located in cavities distributed throughout the biomolecular surface, which exhibit specific physicochemical and geometric properties and ultimately, dictate the preference for molecules to bind into them (3,4). Thus, the detection and characterization of biomolecular cavities play an important role in rational structure-based drug discovery and design pipelines. Based on this, several *in silico* methods have been developed to detect and describe binding sites, which are usually classified as evolutionary-, energy- and geometry-based methods (1,3). Unfortunately, these methods can be computationally intensive enough to hinder users who do not have local access to the necessary computational resources.

In recent years, web services communicating via HyperText Transfer Protocol (HTTP) web protocols have become increasingly popular in cloud computing environments. These services provide extensive access to data and processing resources (https://www.w3.org/TR/wsdl/). Several web services have been proposed for the detection and/or characterization of binding sites in biomolecules. Amongst them, we could cite FpocketWeb (5), GHECOM (6), CaverWeb (7), CASTp (8), ConCavity (9), Depth (10), MoloVol (11) and 3DLigandSite (12).

Recently, we have developed parKVFinder (13), a robust method for detecting and characterizing binding sites in biomolecules, using a geometric grid-and-sphere method and thread-level parallelization. Compared to other software for cavity detection, parKVFinder has an intuitive set of parameters and has been extensively benchmarked in the literature for detection and computational capabilities, delivering accurate and robust performance with any type of protein cavity (1,2,4,13). Although other methods can also detect protein binding sites, each has its specific set of characterizations. parKVFinder stands out by combining geometric and physicochemical characterizations of binding sites, effectively aiding users to identify functionally relevant cavities and to study the molecular recognition process.

Aside from parKVFinder's advances in performance and usability, the installation and configuration procedures of our software, as well as other standalone cavity detection software, still pose a major barrier to users who lack the technical knowledge ideally required to perform them. Furthermore, scientists, educators, and students may not have the local computational resources required for the calculations, which can affect the proper use of cavity detection and characterization methods. In this scenario, we introduce KVFinder-web, an open-source web-based application of the parKVFinder software, consisting of a RESTful web service for cavity detection and characterization and a graphical web portal. Our web application aims to democratize and expand even further the parKVFinder user base in the structural biology community. These users will be able to perform cavity detection on third-party computing platforms, such as institutional servers or cloud infrastructures, using parKVFinder as a service (SaaS). This can also benefit end users worldwide who lack either the necessary local computing infrastructure or technical skills.

## METHODOLOGY

KVFinder-web is a powerful web-based tool designed to detect cavities in a wide range of biomolecular structures, including but not limited to proteins and nucleic acids. It operates on a standard client-server architecture, which comprises two independent components: a graphical web portal and a RESTful web service. The interactive web interface (KVFinder-web portal) provides users an easy-to-use platform to execute the parKVFinder software and analyze the results through any web browser. Supported browsers and operating systems are available in the Supplementary Material. Meanwhile, the web service (KVFinder-web service) itself executes parKVFinder (13) with its geometric grid-and-sphere-based method, as detailed in (2,4,13). To further enhance user experience, we also provide alternative client-side applications, including a Python HTTP client and a graphical PyMOL plugin (PyMOL KVFinder-web Tools). These provide users with additional ways to interact with the web service and analyze their results. To ensure more comprehensive characterization, KVFinder-web has been integrated with additional features, such as depth calculation and Eisenberg & Weiss hydropathy characterization, which are implemented in pyKVFinder (4). These features are now part of the latest version of parKVFinder (v1.2.0) and enable users to obtain more robust and detailed results.

### KVFinder-web portal

**Figure 1.**
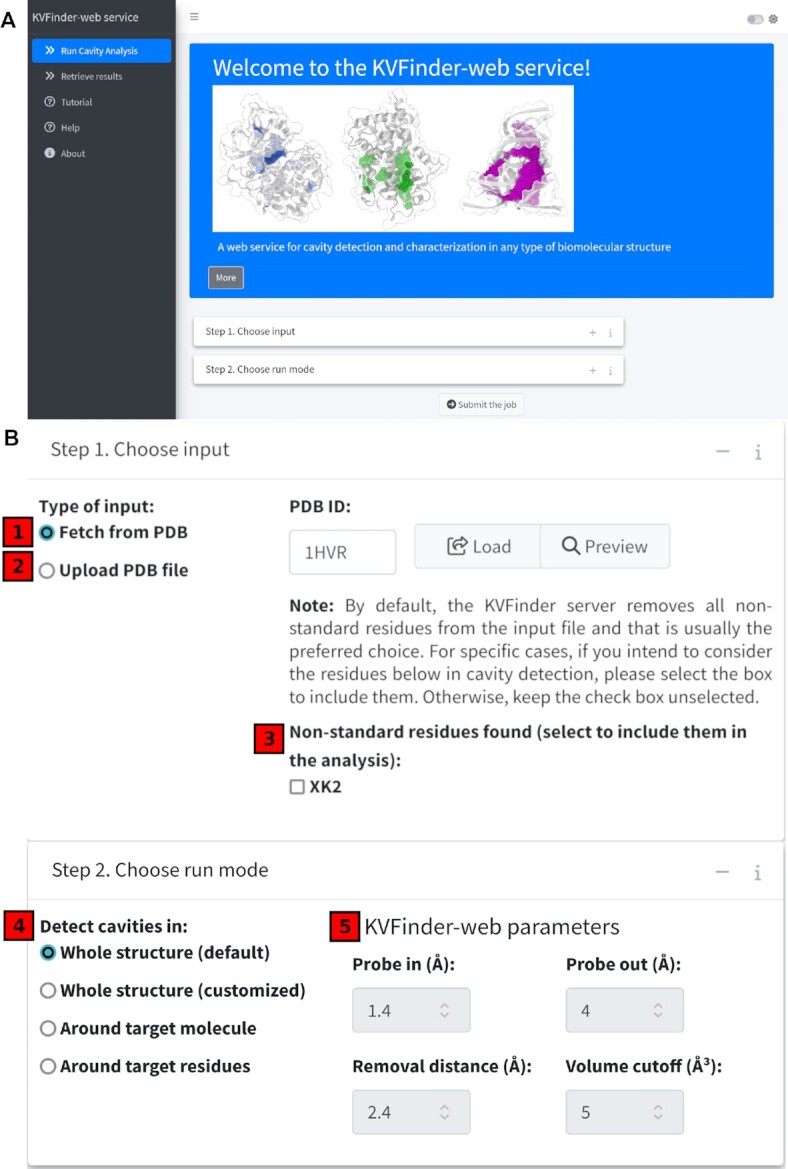
KVFinder-web portal. (**A**) Screenshot of the KVFinder-web portal main page showing the main tabs and the sections of target biomolecule input and choice of KVFinder-web run mode. (**B**) Detailed view of each step that users must complete before submitting the target biomolecule for cavity analysis. The first step comprises the selection of the target biomolecule which can be done by providing a PDB ID and fetching from the PDB database [1] or by uploading a PDB file [2]. After uploading the PDB, the KVFinder-web portal checks the PDB and informs non-standard residues detected [3]. In the next step, users must select an appropriate run mode [4] and customize, if necessary, the detection parameters [5].

The KVFinder-web portal (Figure 1; https://github.com/LBC-LNBio/KVFinder-web-portal), developed using the Shiny R package (14) (https://shiny.rstudio.com), provides a simple and straightforward website for cavity analysis and visualization, requiring only a biomolecule in Protein Data Bank (PDB) format or PDB ID. The web interface is organized into five tabs, entitled ‘Run Cavity Analysis’, ‘Retrieve results’, ‘Tutorial’, ‘Help’ and ‘About’ (Figure 1A).

#### Cavity analysis

The ‘Run Cavity Analysis’ tab features the major functionalities of KVFinder-web, in which users can load a target biomolecule, customize the cavity detection parameters (‘Probe In’, ‘Probe Out’, ‘Removal Distance’, and ‘Volume Cutoff’), and download and visualize results. To execute KVFinder-web (Figure 1B), users must first choose a biomolecule and then select an appropriate run mode.

##### Step 1. Choose input

KVFinder-web allows users to submit any kind of target biomolecule for cavity analysis (Figure 1B). This can be done by uploading a file containing a structure in PDB format or by informing a four-digit PDB ID. In both cases, the input will be checked for non-standard protein or nucleic residues, e.g. water, ligands, and ion molecules, and by default, these will be removed in further steps. However, users can use the ‘Include’ option to keep the non-standard residues in the cavity analysis. The fetching process and biomolecule structure preprocessing are performed internally using the Bio3D R package (15).

##### Step 2. Choose run mode

Four cavity detection modes are available (Figure 1B), to provide options that best suit to the users' cavity analysis, that are:

- Whole structure (default): detection of cavities throughout the whole biomolecular structure with preset detection parameters.
- Whole structure (customized): detection of cavities throughout the whole biomolecular structure with customizable detection parameters.
- Around target molecule: detection of cavities within a radius of each atom of a ligand or molecule in the target biomolecular structure with customizable detection parameters.
- Around target residues: detection of cavities within a custom box, which is drawn by selecting residues of the target biomolecular structure and box padding.

In each run mode, in order to optimize the cavity detection, except for ‘Whole structure (default)’, the user can customize a set of detection parameters that include ‘Probe In’ size (Å), ‘Probe Out’ size (Å), ‘Removal Distance’ (Å) and ‘Volume Cutoff’ (Å³) (Figure 1B). Briefly, the ‘Probe In’ consists of a smaller probe that rolls around the target biomolecule, defining its molecular surface (usually defined as a sphere the size of a water molecule – 1.4 Å), while the ‘Probe Out’ is a larger probe that rolls around the target biomolecule, defining inaccessibility regions. Thus, cavities are defined as the regions accessible to Probe In, typically more inclusive, but not to Probe Out. ‘Removal Distance’ is a length (in Å) removed from the cavity-bulk boundary to delineate the outer limits of the cavity. ‘Volume Cutoff’ is a cavity volume filter (in Å³) to exclude cavities with volumes smaller than this cutoff, probably functionally irrelevant cavities. For a more detailed explanation of each parameter, see (2,4,13).

**Figure 2.**
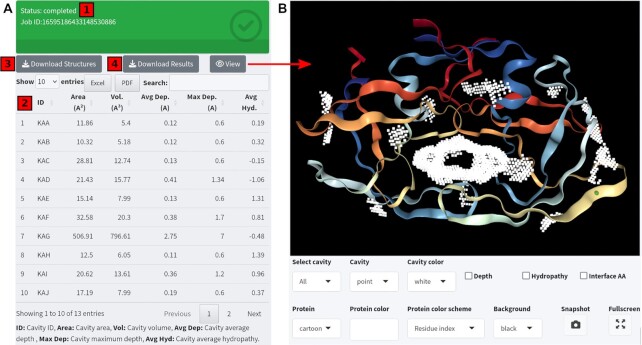
Results and visualization in KVFinder-web portal. (**A**) Screenshot of the results section of the KVFinder-web portal. A box highlights the status (green: ‘completed’; yellow: ‘running’ or ‘queued’; red: ‘canceled’) of the submitted job [1]. Upon completion, the web portal presents results in a data table, including volume, area, average depth, maximum depth and average hydropathy of the cavities [2]. Users can download a ZIP file, containing the target biomolecule and cavity PDB files [3] or a TOML file with cavity characterization [4]. (**B**) The target biomolecule with the detected cavities can be visualized upon clicking on the ‘View’ button and users can customize the visualization of the biomolecule and cavities.

After completing these two steps, users submit the job to the KVFinder-web service by clicking the ‘Submit the job’ button. After successful submission, the web portal automatically updates a unique job ID and a ‘Check Results’ button on the screen. By clicking on the ‘Check Results’ button, the interface displays the current job status - ‘queued’, ‘running’ or ‘completed’ - together with the respective results when available (Figure 2A).

##### Step 3. Results and Visualization

The KVFinder-web portal provides an easy and interactive way to download and visualize cavity detection results. For each cavity detected, the following properties are available to the user: three spatial cavity properties (volume, area and depth), and one physicochemical property (hydropathy) (Figure 2A). Users have options to download the results as PDB files that can be visualized in biomolecular visualization programs, e.g. PyMOL (16), ChimeraX (17), NGL Viewer (18,19) and VMD (20), and download characterizations as a TOML file containing the volume, area, average depth, maximum depth, average hydropathy and biomolecule residues that compose the cavity, as well as the parameters used to perform the cavity analysis (Figure 2A).

Users can also visualize the biomolecule 3D structure with the detected cavities by clicking on the ‘View’ button. The biomolecule viewer uses the NGL engine for R (https://github.com/nvelden/NGLVieweR) (18,19) and provides users with various editing options to customize the visualization style (Figure 2B). These options include selecting target cavities, zooming in on them, showing residues around the cavities, and changing cavity and protein colors, and cavity and protein representations. These features enable users to generate high-resolution snapshots that are well-suited for cavity visualization in publications. Furthermore, the depth of each cavity point, stored as temperature factor (B-factor), can be visualized by clicking on the ‘Depth’ checkbox, the hydropathy, stored as occupancy factor, by clicking on the ‘Hydropathy’ checkbox, and the interface residues, by clicking on the ‘Interface AA’ checkbox.

##### Retrieve results

Users who have already submitted a job can check the job status, using the job ID they receive upon submission, and if completed, retrieve the cavity analysis. This tab provides options for viewing the results online or downloading the result files. Nevertheless, users may share their jobs with colleagues by job ID using this tab.

##### Tutorial, help and about

The ‘Tutorial’, ‘Help’ and ‘About’ tabs contain tutorial, documentation and information about development and our cavity detection suite.

#### KVFinder-web service design

The KVFinder-web service (https://github.com/LBC-LNBio/KVFinder-web-service) is a RESTful service with a Web-Queue-Worker architecture. The ‘Web server’ module, developed in Rust using the Actix framework (https://actix.rs), receives job requests in JSON format via HTTP POST. The received data must contain the molecular structures and parKVFinder detection parameters. The molecular structure of a target biomolecule and optionally a ligand or molecule are stored in the JSON keys ‘pdb’ and ‘pdb_ligand’ and the values are the concatenation of PDB file lines into a single string. If the HTTP request is valid, the web server returns a response with a unique job ID, created using a hash function based on the received data of the incoming JSON. Otherwise, it returns an HTTP error code with an error message. For more information on the JSON data structures, see Supplementary Material.

Jobs accepted by the ‘Web server’ module are sent to the ‘Queue’ module, an instance of the job queue server Ocypod (https://github.com/davechallis/ocypod), where they wait to be processed by a ‘Worker’ module. The ‘Worker’ module communicates with the ‘Queue’ module and requests the next job to be processed by the parKVFinder software. After the job is completed, the cavity analysis (cavities and their characterization) is sent back to the ‘Queue’ module, updating the job status and results, which are made available to the clients via the ‘Web server’ module. To retrieve the job results, the client sends an HTTP GET request with a job ID and the Web server returns the current job status - ‘queued’, ‘running’ or ‘completed’ - along with the respective results, if available. Each job remains cached in the queue for one day after its completion (v1.1.0). Since the job ID is created by applying a hash function on the received data, the cached results are returned when the request is resubmitted.

Each KVFinder-web service module is packaged in a Docker container (21), making it available to run in local or cloud computing environments. These modules are combined in a Docker Compose file for ease of deployment. By default, only one worker is started, but the architecture is designed to allow multiple workers to run concurrently. Besides our publicly available KVFinder-web service, a local web service can also be configured on third-party platforms, such as institutional servers or cloud services, using parKVFinder as a service (SaaS).

##### Limitations

The KVFinder-web service has some limitations compared to a standalone version of parKVFinder, which are designed to prevent very demanding jobs from exhausting the web service. Therefore, some parKVFinder parameters are constrained or even predefined.

##### Future work

The KVFinder-web application components (KVFinder-web service and KVFinder-web portal) will be continuously improved and updated to meet the needs of the scientific community, including new characterizations and performance enhancements.

#### Client-side applications

In addition to the primary interaction mode of KVFinder-web, we also provide additional client-side applications, such as PyMOL KVFinder-web and an example of a Python HTTP client, which broaden the range of possibilities for user interaction.

##### PyMOL KVFinder-web tools

For users familiarized with PyMOL (16), PyMOL KVFinder-web Tools (Supplementary Figure S1; https://github.com/LBC-LNBio/PyMOL-KVFinder-web-Tools), developed in Python3 and Qt, integrates the KVFinder-web service with the molecular visualization software. This user-friendly graphical user interface (GUI) enables customization of detection parameters for a target biomolecular structure and submits jobs to a configured KVFinder-web service (Supplementary Figure S1A). As in parKVFinder PyMOL plugin (13), the search space can also be adjusted to a custom box (box adjustment mode) and/or a radius within a target ligand or molecule (ligand adjustment mode), instead of detecting and characterizing cavities throughout the biomolecular surface (whole structure mode). After successful submission, accepted jobs are routinely and asynchronously requested to the KVFinder-web service. When a job is completed, the plugin automatically processes the incoming data, cavities and characterizations, to local files and makes them available in the GUI. This graphical plugin operates in a similar way to KVFinder-web portal, the characterizations are shown in lists (Supplementary Figure S1B) and the cavities customized by their properties in PyMOL viewer (Supplementary Figure S1C and D). Nevertheless, submitted jobs in the KVFinder-web portal can be loaded in PyMOL KVFinder-web Tools and vice versa.

##### HTTP client

The KVFinder-web portal and PyMOL KVFinder-web Tools may not fully fulfill the needs of advanced users. For this reason, we developed an example of Python HTTP client script, written in Python3, to guide other developers to create standalone clients according to their specific needs. This client operates using standard parKVFinder input syntax and provides tools to interact with a configured KVFinder-web service.

### RESULTS

The KVFinder-web service is publicly available at https://kvfinder-web.cnpem.br, which is free and open to all users without login requirement.

#### Case study

**Figure 3.**
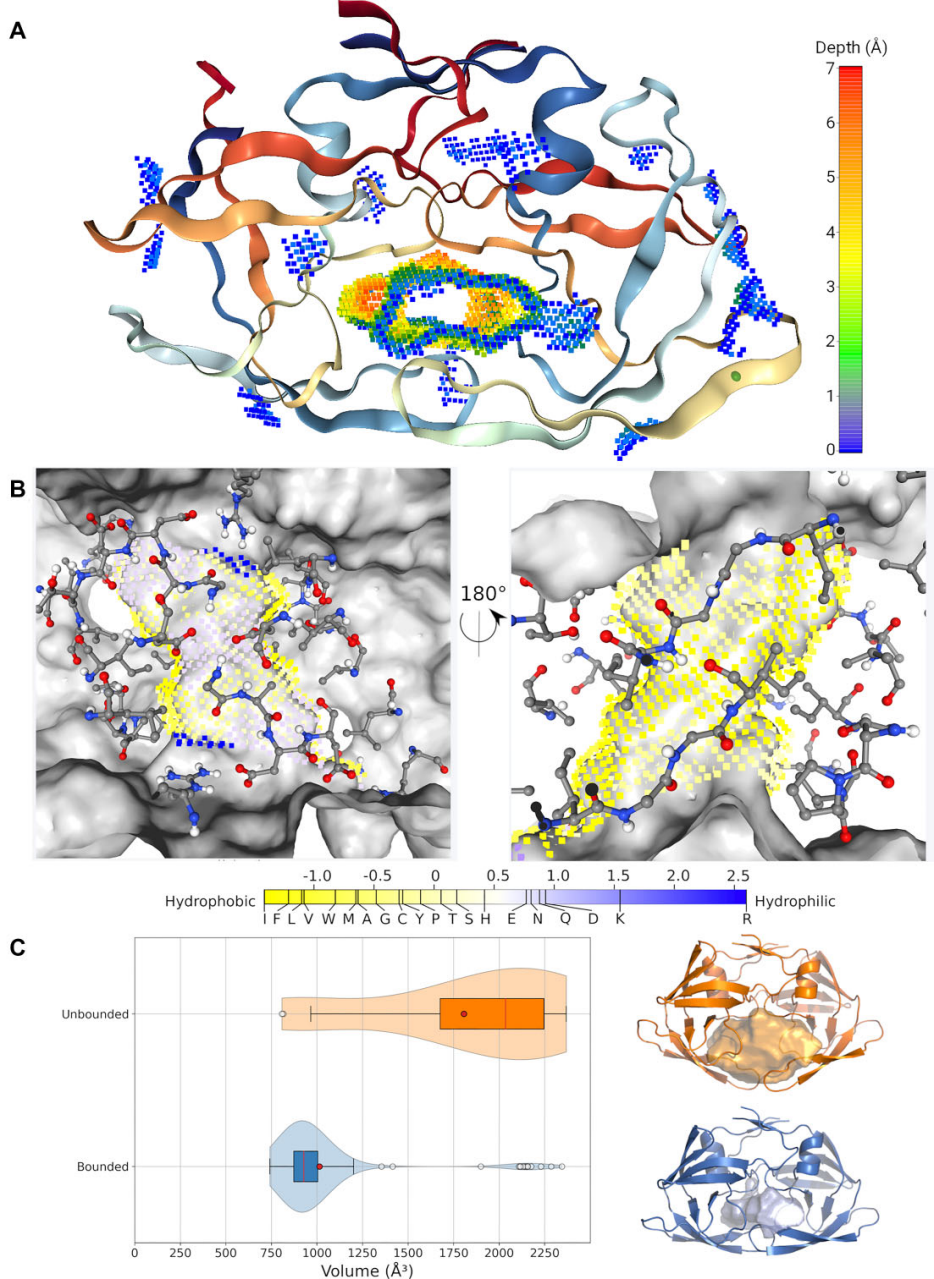
Illustrative example of HIV-1 protease cavity detection. Molecular structure of HIV-1 protease (PDB ID: 1HVR) with detected cavities. (**A**) Cavity detection throughout the protein structure (cartoon). Cavity points are colored by depth (points colored in rainbow scale). (**B**) Hydropathy mapped to surface cavity points in the regions around the catalytic aspartic acids (left panel) and around the β-hairpins (right panel). Eisenberg & Weiss hydrophobicity scale ranges from –1.42 (highly hydrophobic) to 2.6 (highly hydrophilic). Protein is shown as a gray surface and interface residues are shown as atom-colored sticks. (**C**) Violin plot of active site volume of HIV-1 protease structures from the RCSB PDB for the structures with ligands bound in the active site (blue) and the structures without ligands (orange). The structures with a median volume and the corresponding cavity are shown as cartoon and surface, respectively.

As an illustrative example of KVFinder-web (Figure 3), we detected and characterized the catalytic site of the Human Immunodeficiency Virus type 1 (HIV-1) protease bound to cyclic urea carbonyl oxygen (22), using KVFinder-web portal. Since the catalytic site is large and resembles a channel, we adjusted the Probe Out size to 12 Å. Thus, we successfully detected cavities throughout the molecular surface of the HIV-1 protease (Figure 3A). Besides cavity detection and shape definition, KVFinder-web also provides the volume, area, depth and hydropathy of the detected cavities (Figure 2; cavity KAG). Often, the active site is the largest and deepest cavity in the enzymatic protein (23), similarly for HIV-1 protease, as shown in Figure 3B. In this sense, depth characterization can aid researchers to identify the active site throughout the molecular surface.

On the other hand, hydropathy characterization gives valuable insights into the types of interaction and the water attractiveness of the binding site. For instance, we have previously compared the hydropathy profile of an amphipathic pocket located between the E1 and E2 domains of alphaviruses (24), which suggested that a chemically similar molecule (possibly, fatty acids) would bind to them. Additionally, we have analyzed the hydropathy profile of the ADRP substrate-binding site of SARS-CoV-2 and a set of homologous proteins (other related coronaviruses and the human macroD1 and macroD2), revealing that the coronaviruses' pockets share a hydropathy profile, while the human proteins present a less hydrophobic profile (4). In this sense, we conducted an analysis of the hydropathy profile of the HIV-1 protease active site (Figure 3C). Our findings showed that the active site is mostly hydrophobic, especially in the region near the β-hairpins, and there are few hydrophilic regions surrounding the catalytic aspartic acids (Asp-25 and Asp-25′). As expected, the S1 and S1' subsites (yellow region) are hydrophobic, and the S2 and S2' subsites are mostly hydrophobic (yellow region), except for Asp-29 and Asp-30 (blue region). Furthermore, we observed that the hydrophobic part of subsites S2 and S2', which accommodate substrate P2 and P2′, respectively, tend to favor aliphatic side chains (22,25,26).

Since HIV-1 protease is an effective therapeutic target, this catalytic site is the target of several antiretroviral drugs. However, the catalytic cycle depends on the movements of β-hairpins, which control the accessibility of substrates to the active site (13,27). Thus, we analyzed the structures of HIV-1 protease available in the PDB and compared their cavities (Supplementary Material). Based on the cavity volumes, we can clearly differentiate structures with bound ligands from those unbounded (Figure 3C), indicating a geometric complementarity between receptor and ligands, together with a physicochemical complementarity, shown by the hydropathy profile discussed earlier.

#### Detection and computational performance

**Figure 4.**
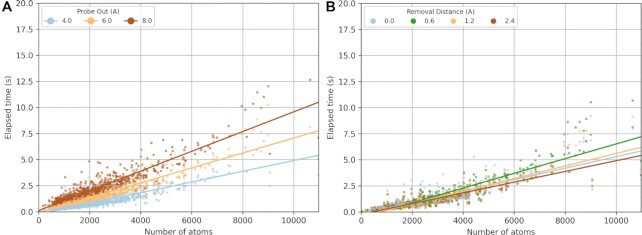
Effects of detection parameters on KVFinder-web service performance. Cavity detection was performed with a protein structure dataset from (13), varying ‘Probe Out’ and ‘Removal Distance’. Elapsed time relative to the number of atoms in the target structure, varying (**A**) ‘Probe Out’ sizes and (**B**) ‘Removal Distance’. The calculations were performed on a desktop computer with an 8-core 3.0 GHz AMD Ryzen 7 1700 processor and 32GB RAM, running Ubuntu 22.04 LTS operating system.

The cavity detection implemented in the KVFinder-web application has been extensively benchmarked against other well-known cavity detection methods in the literature (2,4,13), presenting robust detection and computational performance. In addition, we evaluated KVFinder-web computational performance with a protein structure dataset from (13), varying two major parameters related to cavity detection: ‘Probe Out’ and ‘Removal Distance’ (Figure 4). The systematic translation of ‘Probe Out’ in a Cartesian grid around the biomolecular structure creates a coarser molecular surface, defining the boundary between the cavity and the bulk due to the restricted access to the empty space within the protein. Thus, greater ‘Probe Out’ sizes tend to reduce the degree of accessibility of the molecular surface created and ultimately, increase the elapsed time to perform calculations in KVFinder-web service (Figure 4A). On the other hand, the ‘Removal Distance’ removes cavity points within a given length from the defined cavity-bulk boundary. Thus, reducing the ‘Removal Distance’ removes fewer points from the boundary, which helps to segregate sub-pockets and/or detect superficial cavities. As expected, Figure 4B shows no clear relationship between elapsed time and ‘Removal Distance’, since ‘Removal Distance’ routine is directly affected by the cavity-bulk boundary size and number of cavities, instead of the number of atoms. Finally, independently of these parameters, the execution time increases almost linearly with the number of atoms of the target biomolecule inside the 3D grid. Hence, the number of atoms can be controlled by a custom search box, which can reduce the elapsed time.

### CONCLUSION

KVFinder-web provides simple access to a rapid and accurate method of cavity detection and characterization in any type of biomolecular structure, regardless of the molecule's nature. The KVFinder-web portal provides a simple, easy and straightforward pipeline on any web browser with interactive visualization capabilities powered by NGLVieweR and DataTable. The graphical PyMOL plugin replicates the main features of parKVFinder PyMOL plugin with few limitations to avoid exhausting KVFinder-web service computational resources and to ensure a smooth and efficient operation for all users. Together with the KVFinder-web service, the KVFinder-web portal and PyMOL KVFinder-web Tools aim to democratize parKVFinder software and remove barriers for users who do not have the technical knowledge to install and configure a cavity detection software, who have restricted computational resources, or who just want to perform a simple and quick analysis.

### DATA AVAILABILITY

The publicly available KVFinder-web service is accessible at https://kvfinder-web.cnpem.br. The corresponding open-source code of the KVFinder-web service, KVFinder-web portal, and PyMOL KVFinder-web Tools are available at https://github.com/LBC-LNBio/KVFinder-web-service (https://doi.org/10.5281/zenodo.7825790), https://github.com/LBC-LNBio/KVFinder-web-portal (https://doi.org/10.5281/zenodo.7825788), and https://github.com/LBC-LNBio/PyMOL-KVFinder-web-Tools (https://doi.org/10.5281/zenodo.7825798), respectively. The complete documentation of the modules of the KVFinder-web application is available at https://lbc-lnbio.github.io/KVFinder-web/.

## Supplementary Material

gkad324_Supplemental_File
