# Supplementary material for: KVFinder-web: a web-based application for detecting and characterizing biomolecular cavities
Source: Nucleic Acids Res. 2023 May 4;51(W1):W289–97. doi: 10.1093/nar/gkad324 (PMC10320092; doi:10.1093/nar/gkad324)
Supplement: gkad324_Supplemental_File [file gkad324_supplemental_file.pdf]

SUPPLEMENTARY MATERIAL

Table of contents

Tested browsers and operating systems 2
JSON data structures 3
PyMOL KVFinder-web Tools 5
Cavity analysis of HIV-1 protease 6

Tested browsers and operating systems

We tested the KVFinder-web on multiple operating systems and web browsers. The supported browsers:

- Google Chrome: Windows 10 (21H1), Windows 11 (21H2), macOS Ventura (13.0), Ubuntu (22.04.2 LTS), and Pop!_OS (22.04 LTS);
- Mozilla Firefox: Windows 10, Windows 11, macOS Ventura (13.0), Ubuntu (22.04.2 LTS), and Pop!_OS (22.04 LTS);
- Safari: macOS Ventura (13.0), macOS Big Sur (11.7.4), iOS (16.3.1);
- Microsoft Edge: Windows 10 (21H1) and Windows 11 (21H2).

Please note that KVFinder-web may work on other web browsers and operating systems, but these have not been formally tested.

JSON data structures

The following JSON examples illustrate communication with the KVFinder-web service. These examples are based on KVFinder-web service version 1.1.0 and may change over time as our web service evolves and improves. For detailed documentation of our webservice service, please visit https://lbc-lnbio.github.io/KVFinder-web.

Input


```
{
        "pdb":  "MODEL 1\nATOM 1 N GL.00100.00 N \nATOM ….",
        "settings": {
                "modes": {
                        "whole_protein_mode": true,
                        "box_mode": false,
                        "resolution_mode": "Low",
                        "surface_mode": true,
                        "kvp_mode": false,
                        "ligand_mode": false
                },
                "step_size": { "step_size": 0.0 },
                "probes": {
                        "probe_in": 1.4,
                        "probe_out": 4.0
                },
                "cutoffs": {
                        "volume_cutoff": 5.0,
                        "ligand_cutoff": 5.0,
                        "removal_distance": 2.4
                },
                "visiblebox": {
                        "p1": { "x": 0.0, "y": 0.0, "z": 0.0 },
                        "p2": { "x": 0.0, "y": 0.0, "z": 0.0 },
                        "p3": { "x": 0.0, "y": 0.0, "z": 0.0 },

                        "p4": { "x": 0.0, "y": 0.0, "z": 0.0 }
                },
                "internalbox": {
                        "p1": { "x": -4.0, "y": -4.0, "z": -4.0 },
                        "p2": { "x": 4.0, "y": -4.0, "z": -4.0 },
                        "p3": { "x": -4.0, "y": 4.0, "z": -4.0 },
                        "p4": { "x": -4.0, "y": -4.0, "z": 4.0 }
                }
        }
}
```

Output (queued)


```
{
	"id": "4990580026958948484",
	"status": "queued",
	"output": null,
	"created_at": "2023-03-03T18:55:28.439300871Z",
	"started_at": null,
	"ended_at": null,
	"expires_after": "1day"
}
```


Output (running)


```
{
	"id": "4990580026958948484",
	"status": "running",
	 "output": null,
	 "created_at": "2023-03-03T18:55:28.439300871Z",
	"started_at": "2023-03-03T18:55:31.416200437Z",
	"ended_at": null,
	"expires_after": "1day"
}
```


Output (completed)


```
{
	"id": "4990580026958948484",
	"status": "completed",
	"output": {
	    "pdb_kv": "MODEL 1\nATOM 1 HA KAA 259 -6.711 -11.125 -11. ...",
	    "report": "# TOML results file for parKVFinder software\n ...",
	    "log": "==========\tSTART\tRUN\t=========\n\nDate and tim ..."
	 },
	"created_at": "2023-03-03T18:55:28.439300871Z",
	"started_at": "2023-03-03T18:55:31.416200437Z",
	"ended_at": "2023-03-03T18:55:34.701908960Z",
	"expires_after": "1day"
}
```

PyMOL KVFinder-web Tools

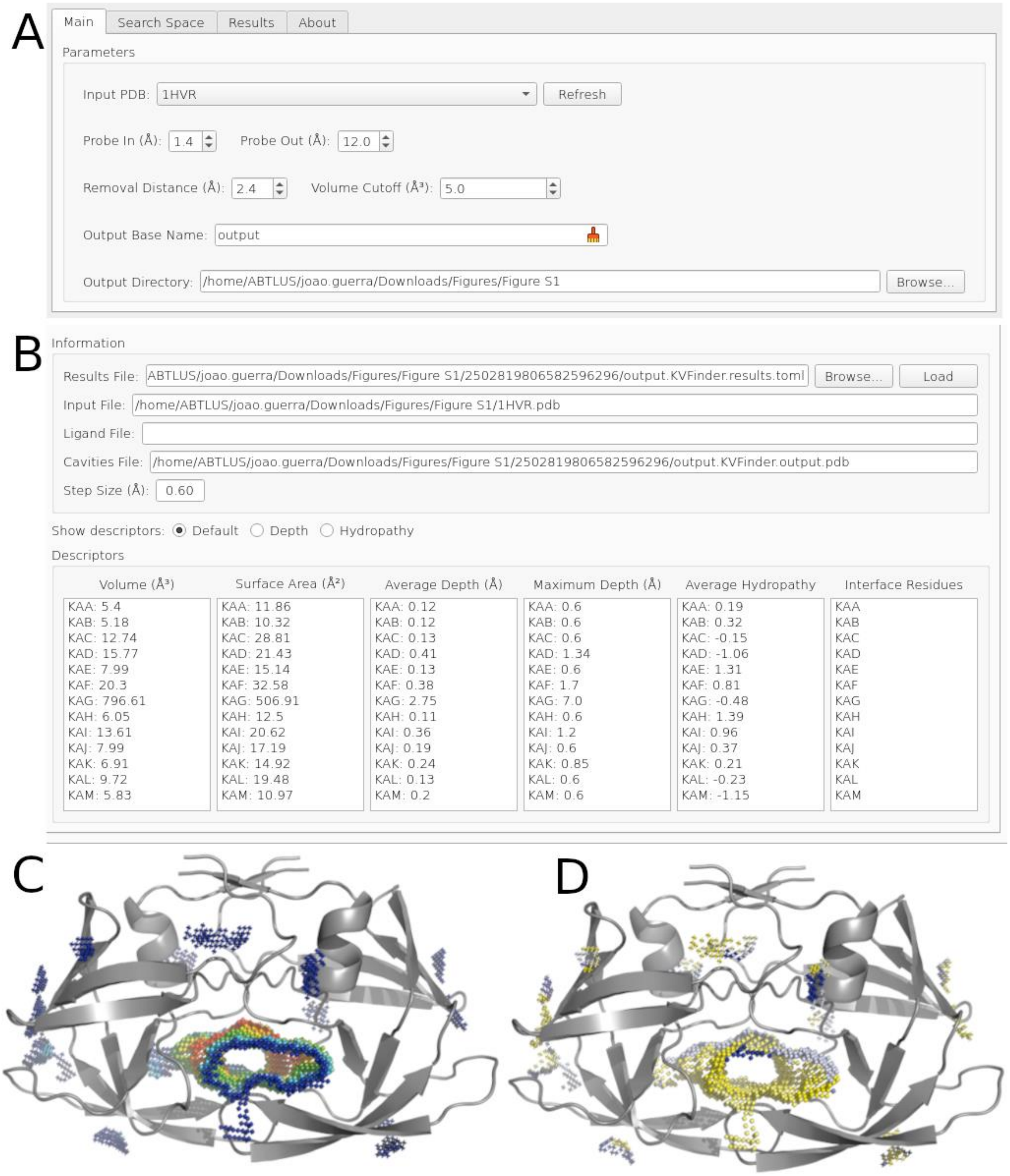

Figure S1: PyMOL KVFinder-web Tools. Cavity detection in the structure HIV-1 protease (PDB ID: 1HVR), with "Probe Out" set to 12 Å. (A) Main parameters tab containing detection parameters and molecular structures to be explored. (B) Visualization tab containing incoming data (cavities and characterizations) from KVFinder-web service displayed in the GUI. (C) Depth characterization and (D) Eisenberg & Weiss hydropathy characterization, highlighting the active site (cavity KAG) in the GUI and PyMOL viewer.

Cavity analysis of HIV-1 protease

As of March 1, 2023, there were 233 X-ray structures of HIV-1 protease available in the RCSB PDB database (https://www.rcsb.org/). Out of these structures, 219 had ligands bound in the active site while 14 did not have any ligands. To prepare the structures for analysis, we removed water, ions, and solvent molecules, extracted the ligands from the receptors, and aligned all the structures. The dataset is available at Zenodo: https://doi.org/10.5281/zenodo.7698665. With that, we detect their cavities with "Probe Out" of 12 Å in KVFinder-web service, interacting with a Python HTTP script. These calculations were performed on our publicly available KVFinder-web at https://kvfinder-web.cnpem.br.
